# Stability and Responsiveness in a Self-Organized Living Architecture

**DOI:** 10.1371/journal.pcbi.1002984

**Published:** 2013-03-28

**Authors:** Simon Garnier, Tucker Murphy, Matthew Lutz, Edward Hurme, Simon Leblanc, Iain D. Couzin

**Affiliations:** 1 Department of Ecology and Evolutionary Biology, Princeton University, Princeton, New Jersey, United States of America; 2 Department of Zoology, University of Oxford, Oxford, United Kingdom; Universite de Toulouse, France

## Abstract

Robustness and adaptability are central to the functioning of biological systems, from gene networks to animal societies. Yet the mechanisms by which living organisms achieve both stability to perturbations and sensitivity to input are poorly understood. Here, we present an integrated study of a living architecture in which army ants interconnect their bodies to span gaps. We demonstrate that these self-assembled bridges are a highly effective means of maintaining traffic flow over unpredictable terrain. The individual-level rules responsible depend only on locally-estimated traffic intensity and the number of neighbours to which ants are attached within the structure. We employ a parameterized computational model to reveal that bridges are tuned to be maximally stable in the face of regular, periodic fluctuations in traffic. However analysis of the model also suggests that interactions among ants give rise to feedback processes that result in bridges being highly responsive to sudden interruptions in traffic. Subsequent field experiments confirm this prediction and thus the dual nature of stability and flexibility in living bridges. Our study demonstrates the importance of robust and adaptive modular architecture to efficient traffic organisation and reveals general principles regarding the regulation of form in biological self-assemblies.

## Author Summary

While migrating, the nomadic army ant *Eciton burchellii* forms long trails of workers that can extend over hundreds of meters in the rain forest. To facilitate the movement of sometimes millions of individuals on uneven and unpredictable terrains, part of the ant workers link together their legs and bodies to form temporary bridges over gaps along the trails. In this work we showed that these bridges were formed readily when the flow of ants hit an unspanned gap and were dismantled very quickly after traffic has ceased on the trail. However, we also observed that the bridges were formed and remained stable under a large spectrum of the traffic intensities on the trail. Using field experiments and computer simulations we discovered the construction rules used by the ants to create these living structures that are capable of enduring variations of the traffic while remaining highly responsive to its interruption. These results offer important insights about the mechanisms that regulate biological self-assemblies and they have potential applications in swarm robotics and swarm intelligence.

## Introduction

The functional complexity of many collective biological organisations, including aggregates of cells [1], tissues [2], [3] and organisms [4]–[6], derives from the integration and processing of information between system components. Tissue and organ functions emerge from mechanical, chemical and/or electrical communication among cells, and many collective activities of group-living animals result from relatively locally-mediated interactions [4]–[8]. Collective dynamics are also prevalent in human societies; networks of social interactions influence a wide range of social processes such as adoption of health behaviour [9], the spread of opinions [10], [11], gossip [12] and the spontaneous formation of lanes, jamming and oscillatory flows in pedestrian crowds [13]–[16].

Effective traffic organisation is not only central to the success of many modern human societies – it is a crucial feature of coordinated action in many social insects [17]–[20]. For example under crowded conditions, the black garden ant Lasius niger maintains an optimal rate of food return by either spontaneously dividing the traffic on their foraging trails between different routes [18], or by alternating clusters of inbound and outbound ants when no other route is available [19]. Near-blind army ants of the genus *Eciton* offer some of the most spectacular examples of behavioural and physical adaptations to minimise congestion, and to regulate traffic flow. They live in large colonies (on average 700,000 ants [21]–[23]), and live their lives at a high tempo; *Eciton burchellii* workers, despite being approximately 1 cm long, form vast dendritic trail networks that can extend over 100 m with individual ants maintaining speeds of 13 cm s^−1^ [17] (8 cm s^−1^ when carrying prey items [24]).

**Figure 1:**
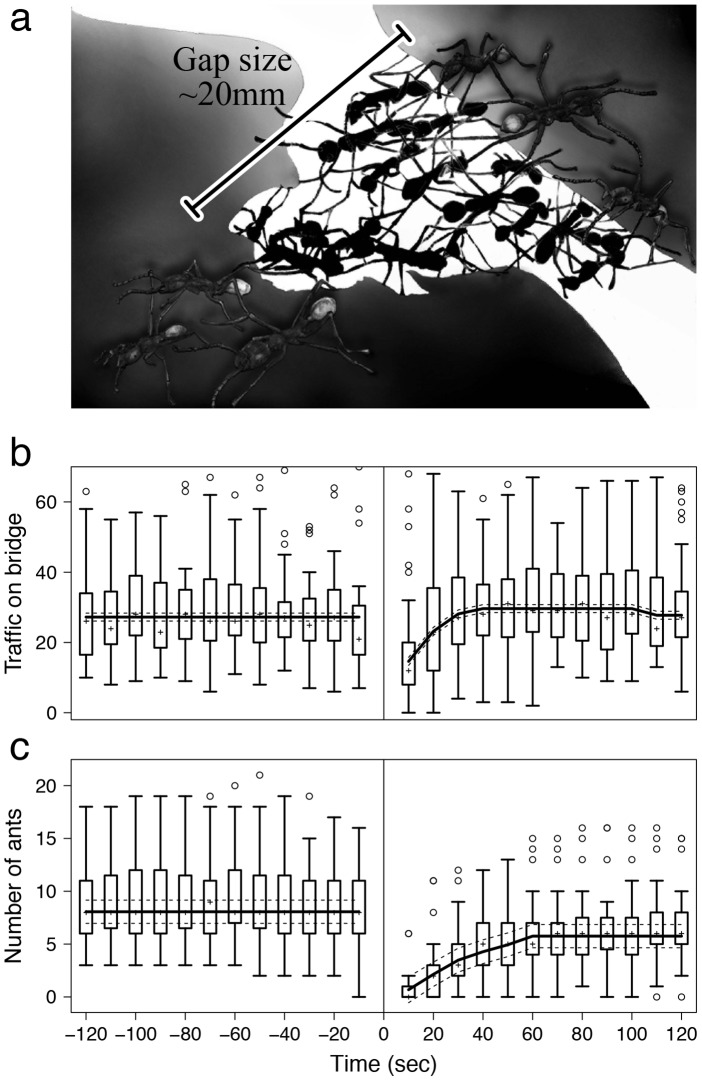
(a) Drawing representing a living bridge formed by the army ant ***Eciton burchellii*** between two leaves (by TM). The black bar illustrate how we measured the gap size as the maximum distance spanned by the ants. (b) Temporal dynamics of the traffic over the gap (in ants/10 second period) and (c) of the number of ants participating in the bridge structure (in ants/10 second period) as a function of time, before (negative times) and after (positive times) the removal of the bridge. For each time period, the cross (+) represents the median value for 39 data points; the box represents the 2nd and 3rd quartiles of the value distribution; the whiskers represent the 1st and 4th quartiles; the circles (o) represent outliers. The solid black line shows an estimation of the evolution of each value by a GLMM, with its 95% confidence interval (dotted lines).

Their high rates of traffic flow must be maintained over highly unpredictable and irregular terrain in order to maintain the prey-capture rate required for colony survival [25]. They thus face challenging traffic coordination problems. An important part of their strategy is their capacity to modify their environment using their own bodies as structural components to fill holes, span gaps, pass overhanging banks and direct flow on sharply-angled turns [21]–[26]. These self-assemblages form by ants linking together legs and bodies with special tarsal claws (see Fig. 1a), a morphological adaptation which also allows them to form temporary nests (“bivouacs”) [26].

To date, it is neither clear how these structures form or whether, and if so how, they are regulated. However the response of individual ants to environmental heterogeneities has been investigated [24]. On uneven terrain, individual workers can use their bodies to plug ‘potholes’, remaining still as long as a consistent flow of traffic is maintained over their body. While offering important insights regarding the rules of thumb adopted by ants in filling holes, this study did not consider the spanning of larger gaps that would require the coordination of multiple ants. Recent experiments with fire ants have investigated collective formation of living rafts to survive flooding events [27]. Ants reaching the raft edge, and perceiving water, exhibit a small probability to attach to the edge instead of walking back. This simple rule is sufficient to generate quickly growing rafts during initial moments of flooding that can remain stable for its duration.

Army ant bridges must be consistently produced and remain coherent and functional within widely varying habitats and under variable traffic conditions. Here we employ an integrated experimental and theoretical approach to reveal how these ants, each of which has access only to relatively local sensory information, coordinate such robust, yet flexible, architectures. We identify the individual-level rules employed by ants when constructing and maintaining bridge structures and reveal how these relate to the functional properties of bridges as a collective structure including exceptional versatility, resilience and fault-tolerance.

## Results

### Dynamics of bridge construction

Army ant bridges are highly dynamic entities, reforming very quickly following removal (see Movie S1). We analysed the dynamics of bridge construction using the following procedure: a natural bridge was located on a trail and filmed for a 10 minute interval following which all ants in the bridge were removed with tweezers (the duration of removal being <1 s); consequently the bridge (typically) reforms, a process we filmed for 10 mins before all ants in the bridge were again removed. This process of removal and reconstruction was repeated twice more, such that each experiment contained a baseline period (during which the “natural” bridge was filmed) and a total of three 10 minutes replicates in which the bridge was successively removed and re-construction was filmed. Thirteen of twenty bridges experimented upon in this way were analysed. Of the seven excluded bridges, it was not possible to satisfactorily observe the ants in four cases, and in the three other cases the ants altered the course of flow after the removal of the first bridge, voiding subsequent trials.

The number of ants participating in the bridge structure, as well as the outward and return flow of ants across the bridge, was recorded for 120 seconds, in 10 second intervals, prior to, and after, each removal of the bridge. Flow rate was measured as the number of individuals that fully crossed the gap. Therefore, individuals that entered the bridge structure were not incorporated into the flow rate, while ants voluntarily exiting the bridge were counted in the flow rate. In addition the size of each gap spanned by a bridge was measured as the longest distance between the objects (leaves or branches) to which the ants are attached on each side of the gap (see Fig. 1a). Gap lengths ranged from 7 mm to 26 mm, with an average length of 14.92 mm. We also measured the bridge width as the longest distance between ants along a line perpendicular to the length of the gap. Bridge width ranged from 8 mm to 55 mm, with all but 2 bridges between 8 mm and 18 mm wide. Note that bridge width was never found to have a significant effect in any of the subsequent analysis and therefore will not be considered in the rest of this study.

The traffic during the two minutes before and after the removal of the bridge is shown in Fig. 1b. Since it was stable prior to removal (GLMM, traffic at −120 vs traffic at −120 to −10 sec: N = 936, p>0.05 in every case) we aggregated traffic occurring over a two minute period preceding the manipulation to serve as a baseline with which to compare to traffic flow following bridge removal. After bridge removal traffic recovers quickly, reaching the baseline value after only 30 seconds (GLMM, traffic at 30 sec vs. baseline traffic: N = 936, z = 0.838, p = 0.402). A small but significant excess of traffic occurs from 40 to 100 seconds after bridge removal (GLMM, traffic at 40 to 100 sec vs. baseline traffic: N = 936, z = 4.847, p<0.0001), likely due to the accumulation of ants on each side of the gap. After 110 seconds, traffic resumes its baseline value (GLMM, traffic at 110 to 120 sec vs. baseline traffic: N = 936, z = 0.664, p = 0.507).

The corresponding number of ants forming the bridge both before and after bridge removal is shown in Fig. 1c. As with traffic dynamics, bridge size is stable during the two minutes prior to the removal and thus can serve as a baseline value with which to compare the reconstruction process (GLMM, bridge size at −120 vs bridge size at −120 to −10 sec: N = 936, p>0.05 in every case). The number of ants participating in the structure increases with time after bridge removal, but does not reach the baseline value (GLMM, traffic at each 10-second period vs. baseline traffic: N = 936, p = <0.0001 in every case); after two minutes, the number of ants participating in the structure represents on average 71% of the baseline value. After 30 seconds, which corresponds to the time after which the traffic over the gap has fully recovered, the number of ants participating in the structure represents on average only 43% of the baseline value. This suggests that only a minority of ants in the bridge are actually necessary to handle the traffic. We also tested the effect of three covariates on the number of ants forming the bridge: the global traffic flow over the gap; the proportion of ants returning toward the bivouac; and the gap length. Only the gap length has a significant effect (GLMM: N = 936, z = 2.409, p = 0.016), with an increase of the number of ants forming the bridge with the gap length.

### The behaviour of individual ants in bridges

In order to relate individual behaviours to the dynamic properties of bridges we recorded the times of arrival and departure, and the incoming direction of the ant (from the bivouac or raiding front) and the caste, for all ants that participated in bridge construction during the 2 minutes after each bridge removal. For all ants that did not participate in bridge construction, we recorded the times of arrival and the incoming direction only, the caste being difficult to assess in moving ants due to their high speed and the low shutter speed of the camera. Individual ants participating in the structure of the bridge were classified into four separate castes, using a visual sorting method that Franks [25] developed and demonstrated to be highly accurate.

In addition we calculated the localised flow rate (recorded as the number of passing ants exhibiting observable tactile contact with the body or antennae of that ant) for 50 separate individual ants (10 individuals randomly selected from 5 separate experiments) that left a bridge voluntarily. These individuals were selected among those that joined the bridge after its removal and they were followed until they left the bridge (for a maximum interval of 10 minutes after which a new bridge removal was performed). To ensure that individuals were really within the bridge structure prior to determining flow rate, only ants that were immobile for at least 10 seconds in the bridge were included in this sample. As much as possible, these ants represent the full range of different durations of time spent in the bridge. The position of the observed ants (edge or centre of the bridge), and the number of surrounding ants to which the focal ant appeared connected (i.e., we could observe a direct contact between the legs of the focal individual and the surrounding ants), was also recorded. We considered ants to be in the centre of the bridge if they were surrounded by at least one other ant on each of their right and left side. Otherwise ants were considered to be on the edge of the bridge.

**Figure 2:**
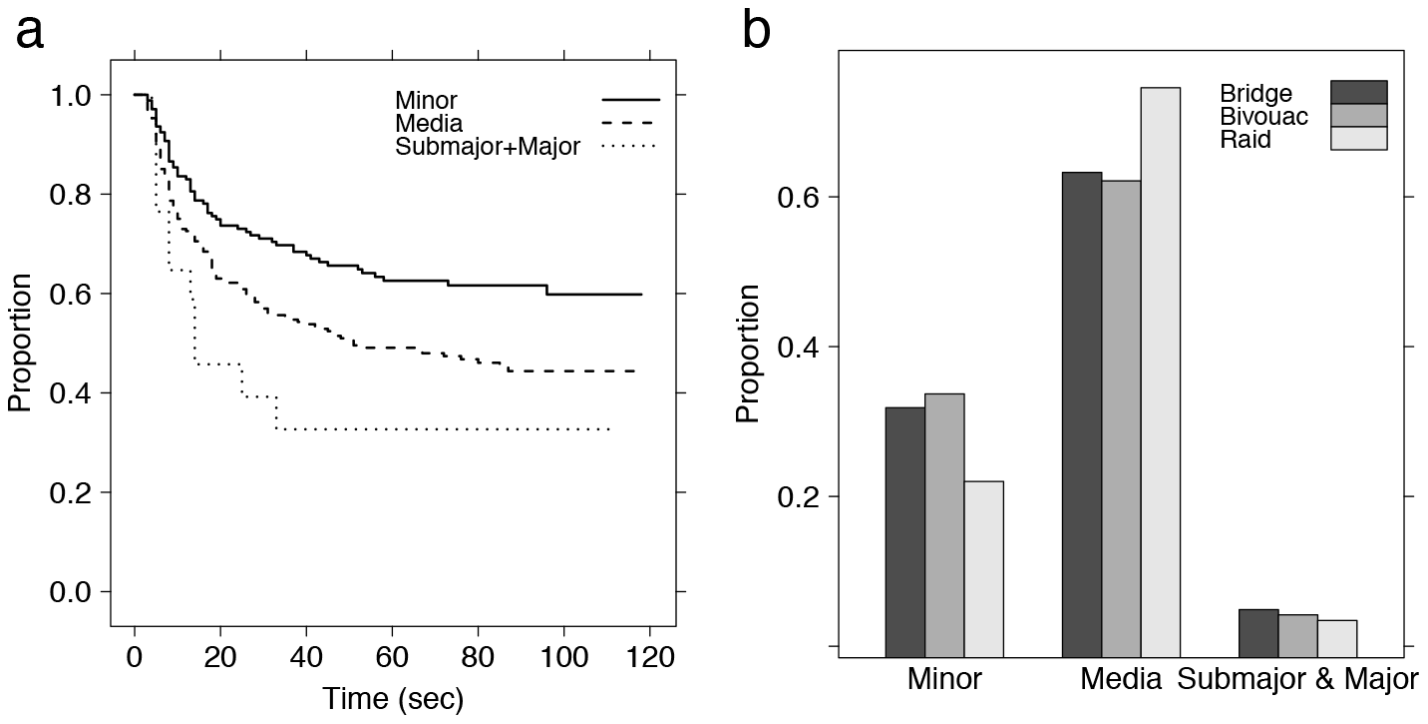
Behavioural differences between castes related to bridge construction. (a) Survival analysis of the time spent by ants as part of the bridge structure during the 2 minutes after the destruction of the bridge. A CPHM reveals that this time decreases with the size (or caste) of the ant as shown here, and increases with the overall packing density of ants on the bridge (number of ants by unit length of the bridge). It also shows that ants joining the bridge earlier are less likely to leave it. (b) Distribution of the different castes across three different dynamic structures (bridge - n = 920 -, bivouac - n = 573 - and raid - n = 3314). The proportion of minor in the bridge is similar to the one found in bivouac, but different from the one found in the raiding trail. The inverse tendency can be observed for media ants. Data for bivouacs and raiding trails from [25].

Ants are more likely to decide to participate in bridge formation if the traffic on the trail (GLMM: N = 15216, z = 2.767, p<0.01) and if the packing density of the bridge (the number of ants participating in the structure divided by the length of the gap) at the instant when the focal ant joins the structure (GLMM: N = 15216, z = −2.671, p<0.01) are lower. The sooner ants participate in bridge formation, the longer they remain as part of the structure (CPHM: N = 449, z = 4.002, p<0.0001). This duration also increases with the packing density of the bridge at the instant when the focal ant joins the structure (CPHM: N = 449, z = −5.337, p<0.0001; see Fig. S1, inset) and is affected by the caste of the ant (see Fig. 2a), minor ants spending more time as part of the structure than media (CPHM: N = 449, z = 2.141, p = 0.0323) and sub-major/major ants (CPHM: N = 449, z = 2.476, p = 0.0133). This suggests a specialization of minor ants in the building of self-assemblage structures, an assumption supported by the over representation of this caste in bridges and bivouacs (see Fig. 2b): the percentage of minor ants in this structures - resp. 31.8% (293/920) and 33.7% (193/573) - is considerably higher than the one found in raiding trails - 22% (729/3314; data for bivouacs and raiding trails from [25]). Note that an opposite tendency is found for media ants (resp. 63.2% - 582/920 -, 62.1% - 356/573 - and 74.5% - 2471/3314). The incoming direction of the ant (from the bivouac or from the raiding front), and the total volume of traffic over the gap, however, do not affect significantly the time spent by ants in the structure of the bridge (CPHM: N = 449, p>0.05 in every case).

**Figure 3:**
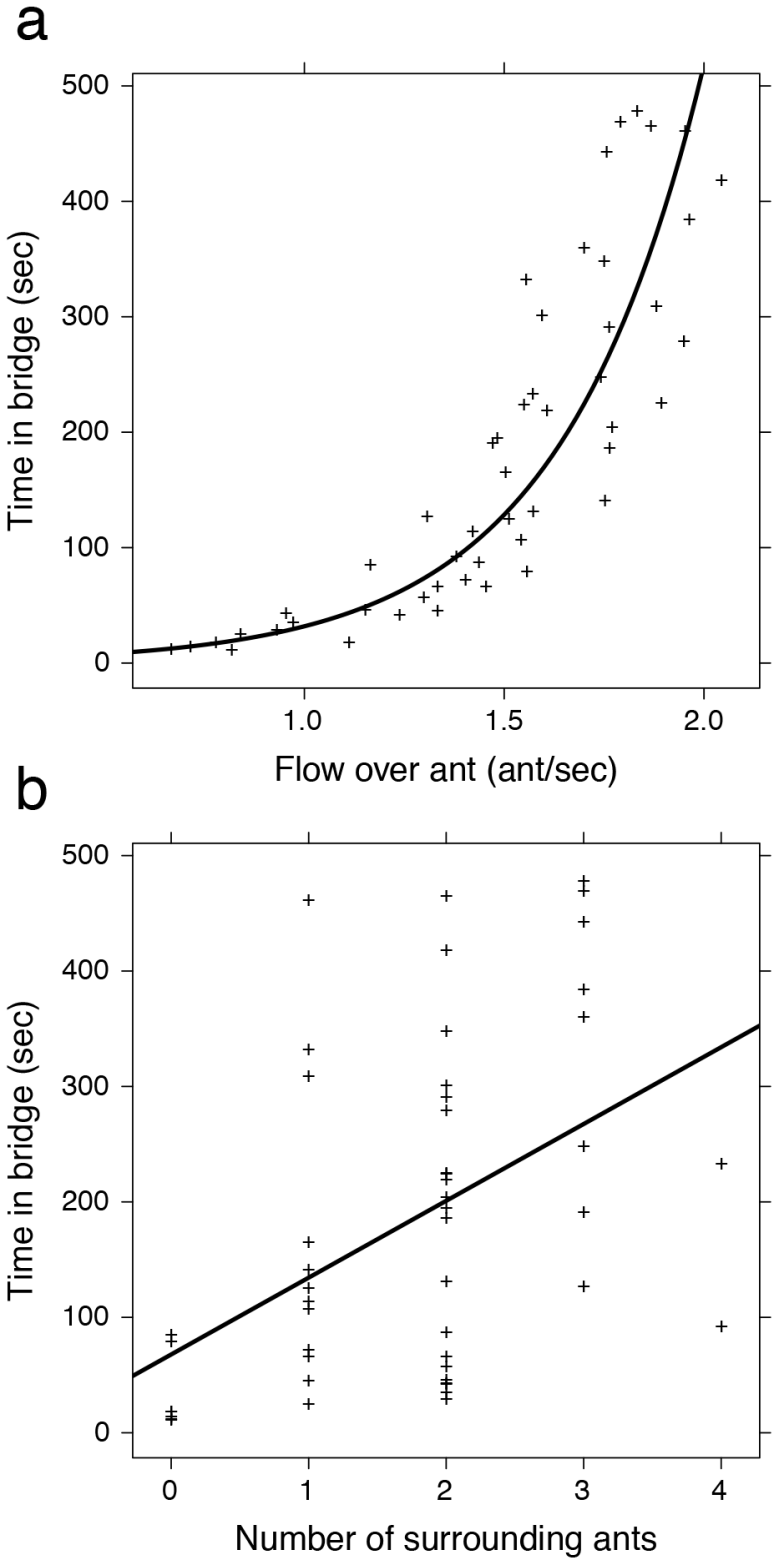
Detailed behavioural analysis of a subset of 50 ants. (a) The time spent in the bridge by an ant ***i*** increases as a function of the traffic flow over this individual (GLMM, p<0.0001). This data is best modeled by an exponential increase of the form 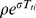, with ***ρ*** = 1.959, ***σ*** = 2.789, and 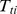 represents the traffic over the individual. (b) The time spent in the bridge by an ant increases as a function of the number of surrounding ants in the structure (GLMM, p = 0.016).

A survival analysis of the time spent by ants as part of the bridge confirms the existence of a stable core of ants, with about 50% of the ants joining a bridge structure remaining part of it after two minutes. The shape of the survival curve suggests that these ants might actually stay in the bridge for much longer periods of time (see Fig. S1), and extended observations of a subset of 50 ants showed that some ants stayed in the bridge for up to 500 seconds (which corresponds to the maximum observation time, see Fig. 3a).

Unlike our previous analysis of the influence of the total volume of traffic, detailed analysis of the aforementioned subset of 50 ants demonstrates that local traffic intensity strongly (and predictably) influences the time an individual remains as part of a bridge (Fig. 3a; GLMM: N = 50, p<0.0001). Furthermore - as suggested by the packing density - as the number of connected ants increases so does the time spent in the bridge (Fig. 3b; GLMM: N = 50, p = 0.016). These variables (local traffic intensity and number of surrounding ants) are significantly correlated (Pearson's product-moment correlation: t = 3.3927, df = 48, p = 0.0014), but removing one or the other variable from the GLMM results in a lower goodness of fit as evaluated with the Akaike Information Criterion and the Bayesian Information Criterion. This suggests that the two effects may be additive. One explanation for the existence of this correlation comes from the spatial organisation of the bridge; ants in the middle part of a bridge experience a significantly higher overhead traffic (see Fig. S2a; W = 137.5, p = 0.0005) and are connected, on average, to a larger number of neighbours (see Fig. S2b; W = 196.5, p = 0.0164).

### Model simulations and bridge response to traffic variations

To formalise the relationship between individual-level behaviour and the dynamics of bridge self-assembly we develop, and analyse, a data-driven, pseudo-spatial individual-based model of the ants' behavior (IBM, see Material and Methods section below for a detailed description of the model). This allows us to validate our understanding and to explore further the stability of the bridge structure under varying traffic conditions, thus allowing us to make explicit and testable predictions.

#### Robustness of ant bridges to typical traffic fluctuations

**Figure 4:**
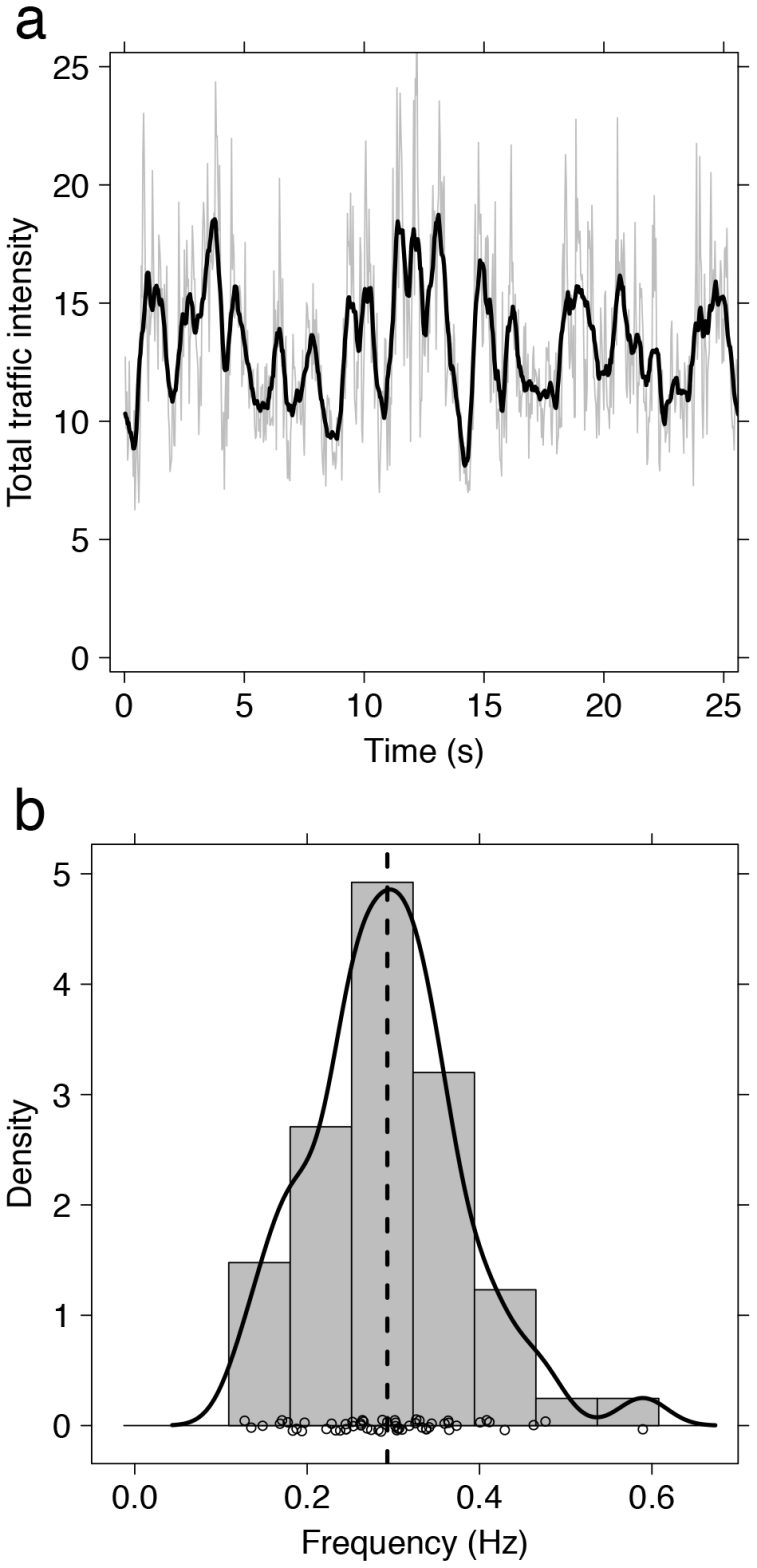
(a) Example of the typical variation in traffic intensity on a foraging trail. Data obtained from the Particle Image Velocimetry algorithm are shown in grey. The black line is obtained by computing a running average on the data with a window of ½ second. (b) Distribution of the dominant frequencies of traffic oscillations on 57 foraging trails. The black circles at the bottom of the figure represent the actual observed frequencies. The grey bars and the continuous black line represent the estimated distribution of these observed densities in the form of a histogram and of a density plot, respectively. The dashed black line represents the median value of the distribution (0.293 Hz, which corresponds to a median period of 3.413 seconds).

In order to investigate how bridges respond to regular traffic conditions we need to determine the nature of typical fluctuations in traffic flow on real trails. We analysed fifty-seven foraging trails using an automated technique employing optical flow ([28]; see Movie S2 for an example and Fig. S3 for comparison with manually tracked data). Army ant traffic exhibits consistent periodic dynamics, an example of which is shown in Fig. 4a. The distribution of dominant frequencies of these oscillations reveals a median period of 3.4 seconds (Fig. 4b). These oscillations likely result from physical interactions and speed differences between the ants, analogous to what happens in vehicular traffic jams [29], [30] or in pedestrian crowds [14], [15].

**Figure 5:**
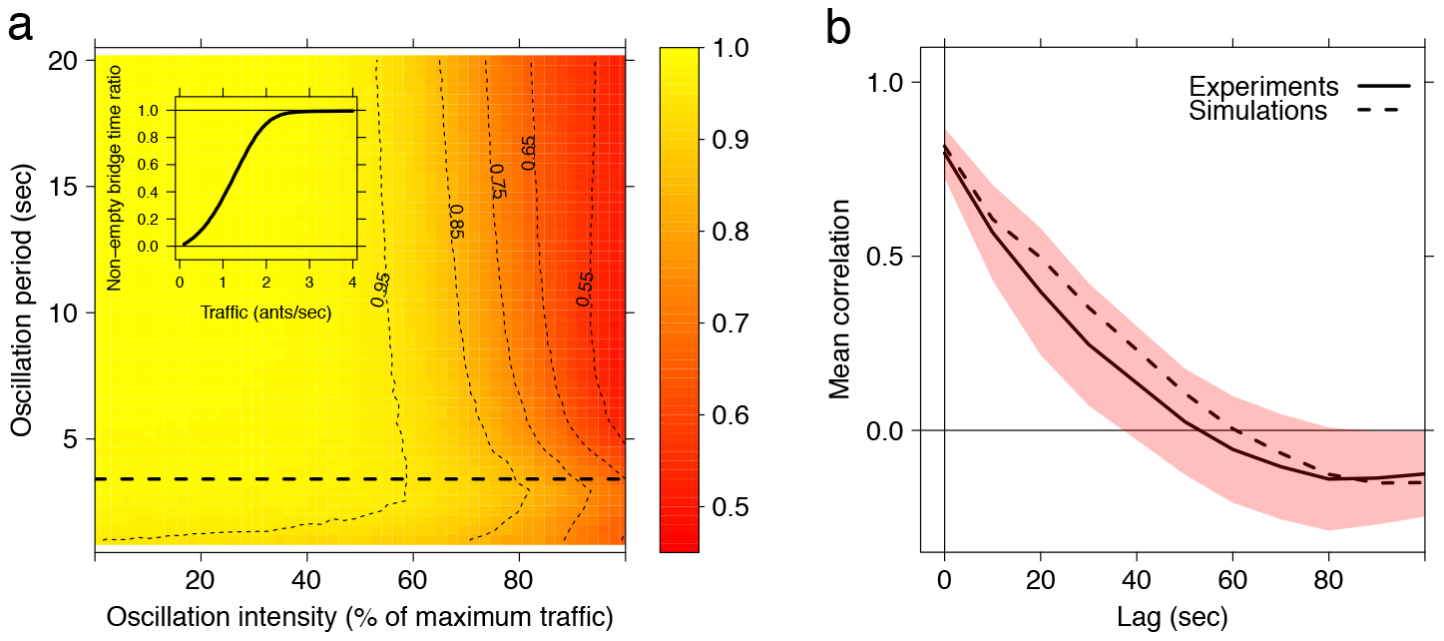
Bridge sensitivity to traffic conditions. (a) Resistance of the bridge to traffic variations. The values represents the non-empty bridge time ratio (***i.e.,*** at least one ant stopped in the gap) as a function of the oscillation period and the oscillation intensity of the traffic on the trail in simulations of the model (simulation length: 100 oscillation periods). The horizontal dashed line represents the typical dominant oscillation period as observed on natural trails of army ants (see Figure 3b). Inset is the non-empty bridge time ratio as a function of the mean traffic flow on the trail in simulations of the model. (b) Bridge reactivity to sudden interruption of the traffic. The solid line represents the mean correlation (the shaded region being the +/−95% confidence interval) between the traffic on the trail (measured by blocks of 10 seconds) and the number of ants bridging the gap during 10 experiments where the traffic was interrupted by sweeping ants off the trail, at different time lags (0 to 100 seconds by 10 seconds intervals). The dashed line represents the same measurement for 10,000 simulations with the same starting conditions (gap size and number of ants bridging the gap when trail sweeping started) and the same traffic conditions as in the experiments.

We tested the effect of the traffic intensity on the ability of the ants to bridge a gap in the parameterized simulations. Construction of a bridge was simulated over a gap of 15 mm (representing the mean gap length of 14.92 mm in the bridge removal experiment). To gain insight into the relationship between traffic intensity and bridge formation we first assume random traffic conditions, with flow rate drawn from a Poisson distribution with parameter λ varying between 0.1 and 4 ants/sec (the range of traffic values observed in the experiments) by increments of 0.05 ants/sec. Computing the mean proportion of time during which the bridge is present (at least one ant spans the gap) for different values of λ we find that the probability of finding a bridge increases nonlinearly as a function of traffic intensity (Fig. 5a, inset). As a consequence, the stability of the bridges does not increase linearly with the intensity of the traffic on the trail; instead, the transition between unstable and stable bridges as a function of the flow rate is closer to being a step function.

We can now ask the following question: how robust are bridges to naturalistic periodic fluctuations in λ? To do so we simulated bridge construction under oscillatory traffic conditions, representing those found in our experimental data (see Fig. 4). Simulated traffic oscillated as a square signal of constant period and intensity; due to the 1 second time step of the simulation using a sinusoidal signal would artificially result in irregular oscillations for short periods. Investigating oscillation periods between 1 and 20 seconds, and flow between 0 and 100% of the maximum traffic intensity (3 ants/sec), we find that the proportion of time during which the gap is not empty decreases with the intensity of the oscillations (see Fig. 5a, moving along intensity axis, from left to right). However this effect does not change in a monotonic way with the oscillation period. A simulated bridge is more likely to form if the oscillations have a period of 3 seconds, whatever the traffic intensity (see Fig. 5a, moving along period axis, from top to bottom). This optimal period is very close to the dominant oscillation period we found in natural ant traffic (3.413 sec; Wilcoxon signed rank test with continuity correction: V = 724, p = 0.4177; see Fig. 5a) and it does not vary with the traffic intensity (if traffic≥2 ants s^−1^, tested up to 5 ants s^−1^) or bridge length (value tested: 5–30 mm).

The stability of simulated bridges decreases non-linearly with the period of the oscillations, providing evidence that bridges act as buffered systems remaining unresponsive to a range of moderate fluctuations and specifically damping the impact on bridge construction of the most frequent forms of inherent traffic oscillations, and therefore stabilizing bridges under normal foraging conditions.

#### Response to sudden changes in traffic intensity

The non-monotonic response to oscillation intensity is suggestive that army ants may have evolved to simultaneously be robust to moderate perturbations (such as those experienced most often) yet remain sensitive to large, sporadic variations in traffic caused by disruptions of the trail (e.g., as resulting from a branch falling on a trail, disturbance through rain [22], or when a trail comes under attack as, for example, through kleptoparasitism of prey items by ant birds [31]) or interruptions in recruitment due to food exhaustion or reduction in light levels [22], [32].

To evaluate their responsiveness to abrupt interruptions in traffic, we performed series of traffic perturbation experiments on an additional 10 bridges. These were filmed for 30 seconds before artificially interrupting the traffic by sweeping ants off the raiding trails on both side of the bridge with small brooms. Sweeping points were established at a minimum distance of 2 meters from either side of the bridge to minimise release of alarm pheromone in the direct vicinity of the bridge. Traffic flow was substantially interrupted by sweeping and filming was stopped when no ant remained bridging the gap (from 80 to 630 seconds after the beginning of the sweeping). Data analysis was performed as described for our previous experiments. We emphasise that the results of traffic perturbation experiment were neither used for model design nor for the estimation of the values of the model parameters.

For each of the 10 natural bridges in the traffic perturbation experiment described above, we also performed 1,000 simulations of our model with the same starting conditions (gap size and number of ants bridging the gap when trail sweeping started) and the same traffic conditions (as measured from the experimental videos). We found a very high degree of congruence between our simulations and experiments, supporting the validity of our model (see Fig. 5b). In both the maximum correlation between the intensity of the traffic (decreasing because of the sweeping of ants off the trail) and the number of ants forming the bridge is positive and obtained for a lag of 0 seconds. Furthermore the values of the maximum correlation obtained from the simulation and experiments are indistinguishable (0.796±0.071 for the experiments, 0.817±0.002 for the simulations, mean ±95% confidence interval). Note also that simulations and experiments are also indistinguishable for all the other tested lags, as shown in Fig. 5b.

Thus bridges, despite being operational under moderate perturbations, remain very responsive to sudden, large perturbations of the foraging activity. Both in experiment and simulation, ants reacted almost instantaneously to the interruption of the traffic on the trail by quickly dismantling the bridge. This selective flexibility is likely to be a powerful asset for army ant colonies, helping them reduce the number of foragers involved in unnecessary structures.

## Discussion

Unlike inert constructions, self-assembling structures have the capacity to rapidly and autonomously adapt their form to changing conditions [33]–[35]. We demonstrate that army ant bridges are versatile and fault-tolerant living architectures whose construction relies on simple rules of thumb. Bridges spontaneously adapt to the physical environment in which the colony operates and exhibit the property of being maximally robust for an oscillation period of approximately 3 seconds (Fig. 5a), whatever the traffic oscillation intensity, a property that stabilises bridges under natural oscillatory traffic fluctuations (Fig. 4a&b). However bridges are simultaneously highly sensitive and responsive in the face of sudden alteration of traffic on the trail. Thus this living architecture filters appropriately variations in traffic, discriminating between normal operating conditions and sudden, large perturbations of the foraging activity. This allows *E. burchellii* colonies to spontaneously create living bridges when and where they are needed, and appropriate to the size and type of gap encountered along the trail. We show that these properties contribute considerably to the efficient operation of *E. burchellii* raiding trails, as do features such as lane formation [17], pothole plugging [24], specialist porter castes [25] and the division of labour among teams of carriers [36], [37].

A detailed analysis of the behaviour of ants participating in bridge formation reveals the individual rules responsible for the above properties. The time spent by an individual in a bridge depends on its connectivity in the structure; highly connected ants being less likely to leave. These data support the view proposed by Schneirla [22] that interconnections among ants induce immobilisation. Highly connected ants are also more likely to occupy a central position in a bridge, where the majority of the traffic occurs thus increasing the stability of the structure and buffering the bridge to moderate fluctuations in flow. Our data suggest that not all the ants forming a bridge are absolutely necessary to ensure efficient traffic flow over a gap. It is plausible that these additional ants may give the bridge capacity to handle sudden increases in traffic.

The time spent by an individual in the bridge was also highly correlated with the caste to which this ant belongs. Ants of larger castes remained in the bridge for significantly shorter periods of time than ants of smaller castes. As a consequence the proportion of ants of each caste found in the bridge was different from the relative fraction of each caste found in the raiding trails [25]. In particular, minims were present in a higher frequency than expected, and medias were found at a lower frequency than expected (submajors and majors were found too rarely in bridges to provide accurate estimations). The relative percentages of each caste most closely approximated that found in *E. burchellii* bivouacs [25], which suggests that the construction principles in bridges and bivouacs are related. The amount of time each caste spent in the bridge appears to correspond with the broader context of their role in the colony. Both the specialist soldier caste (majors [22], [25], [32]) and the specialist porter caste (submajors [25]) spent the least amount of time in bridges. The generalist medium caste [25] stayed in the bridge longer than the specialist castes, though for less time than the minims. The role of the latters in raiding trails is not documented and our results suggest that they could be considered as bridge specialists. However it is not possible to decide whether this specialization is the result of an evolved behaviour. Perhaps, the most parsimonious explanation is that smaller castes have a higher probability of encountering gaps where they are forced to stretch their back legs and trigger their immobilizing mechanism than larger castes. This is supported by recent observations of pothole plugging in the same species showing that larger ants plug larger holes than smaller ants [24]. Therefore, minims' apparent specialization could be a simple side effect of their body length. Regardless of the exact mechanism that leads ants of smaller castes to stay in bridges longer than those of larger castes, minims function like a living pavement that fills in gaps, so that specialist nestmates may efficiently complete their transport and defence tasks.

Army ants within bridges exhibit a strong exponential relationship between the time they remain in the structure and the flow rate over them. The implications of this exponential relationship are considerable for the stability of bridges. For example, an increase in perceived flow rate of approximately 0.25 ants/sec results in doubling the time spent in the bridge. As a consequence, the stability of the bridges does not increase linearly with the intensity of the traffic on the trail. Instead, the transition between unstable and stable bridges as a function of the flow rate is closer to a step function. If the relationship between the flow over an individual and the time that individual spends in a bridge were linear, bridges would be comparatively less stable and less likely to form at high flow rate. The exponential relationship ensures that bridges are rapidly constructed, and are stable under high traffic conditions when bridges are most important to facilitate efficient movement and transportation over the trail network. At low flow rate however, when the maintaining of bridges is more costly relative to the benefits of foraging, this exponential relationship facilitates the disbandment of the unnecessary structure. Therefore, *E. burchellii* can considerably alter the stability of their bridges according to the conditions on the raiding trail.

The highly effective mix of stability and flexibility in this living architecture could provide valuable inspiration for technological solutions in collective robotics or swarm intelligence [38]–[41]. For example the “behavioural algorithm” implemented may be applied to develop self-reconfiguring aggregates of interconnected, or modular robots, or algorithms for dynamic allocation of connection slots in communication networks. Our work also offers insights about the adaptive value and functional design principles of biological self-assemblages [26], [33], [34].

## Materials and Methods

All analysis and statistical tests were performed using R 2.13.0 [42]. Unless otherwise stated, comparisons between two independent samples were performed using the Mann-Withney test and correlations between variables were assessed using the Pearson's product-moment correlation test.

### Analysis of bridge removal experiments

Bridges were filmed on the principal raiding trails of an *E. burchellii* colony during its nomadic phase in Soberania National Park, Panama. Bridges were located within 1–24 meters of the bivouac site and recorded over eight days of the dry season (12th March–20th March, 2001) using a digital video camcorder (Sony DVCAM) between 0900 and 1500 hours.

Analysis of flow over the bridge and the dynamics ants in the bridge were tested with generalised linear mixed effect models (GLMM) with a Poisson error distribution using the “lme4” package (version 0.999375-39). Data were nested as 10-second period per replicate per bridge.

The probability that an ant passing over the gap participate in bridge construction was analyzed with a GLMM with binomial distribution. The time spent by ants as part of the bridge during the 120 seconds after removal were analyzed using the “survival” analysis package (version 2.36-9). A Cox proportional hazards model (CPHM) was fitted to the data, and data were stratified by bridge. The proportional hazards assumption was tested using the method described in [43] and no significant difference from it was found both at a global level and at the level of the different tested factors.

Effects of the localised flow rate and the number of surrounding ants on the behaviour of the subset of 50 ants were tested with a GLMM with a Gaussian error distribution. The validity of the resulting model was verified graphically for each tested factor. P values were obtained using the “pvals.fnc” function from the “languageR” package (version 1.2).

### Optical flow analysis of traffic dynamics

Trails were filmed during stationery and nomadic phases in Sobernia National Park from June 14 to August 10 2009, between 0900 and 1600. At this time of day the flow rate of ants is usually bi-directional. The trails were all located within 50 m from the bivouac and filmed using a high definition camera (Panasonic HDC H300) for periods lasting from 24 to 1370 seconds.

Particle image velocimetry (PIV) [28], an optical flow technique, was used to quantify the flow of ants from a 256×256 pixels window centred on the main axis of trail (the axis being obtained by computing the average variation in intensity for all pixels in the image and fitting a straight line using a weighted regression). The temporal derivative was calculated by differentiating two successive frames, while the two spatial derivatives were calculated using 45 by 45 Sobel filters, and the products of these three derivatives were calculated and convolved with a 100 by 100 Gaussian filter. This resulted in the generation of a ‘velocity field’, i.e. an estimate of the direction and speed of motion for each pixel within the image. By projecting these velocity vectors on the main axis of the trail, and summing the norms of these projections, we could reliably estimate the relative variations of the traffic although due to inherently highly variable lighting and contrast under the rainforest canopy it is not possible to obtain with this method an accurate estimate of the absolute value of the traffic on the trail.

### Quantifying periodicity in traffic flow

We analysed our traffic flow data with Lomb-Scargle periodograms over the frequency range 1/30 to 1 Hz (as described in [44]); statistically significant peaks of the corresponding periodogram for each dataset were averaged following weighting by their spectral power density to reveal the distribution of dominant frequencies.

#### Traffic perturbation experiment

These experiments were undertaken between July 31 and August 9 2011 on Barro Colorado Island, Panama, between 0900 and 1600 and filmed with a high definition camera (Panasonic HDC H300).

### Model

**Figure 6:**
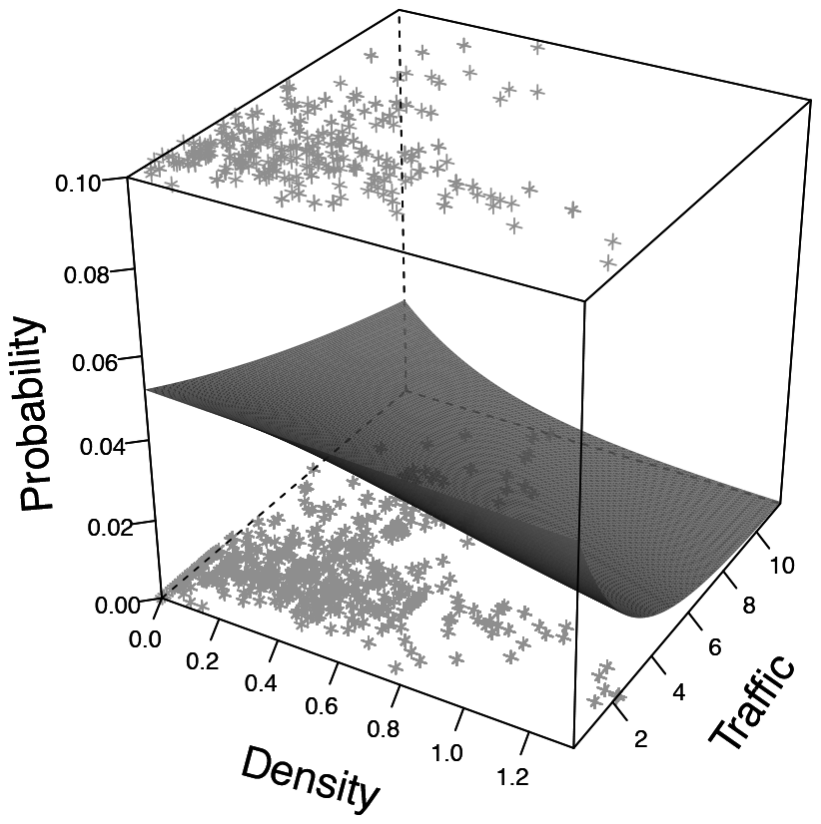
Probability 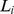 that an ant passing over the gap decides to participate in bridge construction as a function of the packing density of the bridge and the instantaneous traffic on the trail. Light grey stars represent the experimental data; upper stars represent ants that decided to join the bridge structure; lower stars represent ants that crossed the gap without stopping. The grey surface represent the best fit of a 3 dimensional sigmoidal function to the experimental data.

At each time step (1 second, corresponding to the unit of time we used to analyse the individual behaviours of the ants), a random number 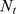 of ants, drawn from a Poisson distribution with parameter λ, cross a gap of a given size. Each ant has a probability 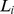 of stopping to form/join the bridge structure. A GLMM on our experimental measurements showed that 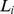 should decrease with the traffic on the trail and the packing density of the bridge, roughly following a sigmoidal decrease in both cases. We chose to model the effect of traffic and packing density on 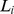 as a 3 dimensional sigmoidal function (see Fig. 6) of the form: 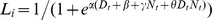, where *D_t_* represents the packing density of ants filling the gap at time t (in ants mm^−1^). *α*, *β*, *γ* and *θ* are control parameters, their values estimated by fitting the previous equation to data of success/failure to join the bridge during the two minutes after the bridge removal (least squares fit: *α* = 0.02; *β* = 144.5; *γ* = 3.258; *θ* = 12.97).

After an ant *i* joins the bridge structure, it exhibits a probability 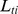 to leave it at time *t*. This probability decreases exponentially with the traffic 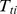 over its body at each time step (*i.e.,* the inverse of the data represented in Fig. 3a): 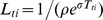. *ρ* and *σ* were estimated by fitting the previous equation to the inverse of the time spent by an ant as part of the bridge as a function of the traffic over its body (Least squares fit: *ρ* = 1.959; *σ* = 2.789).

Finally, the value of the traffic 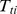 over the body of an ant at time *t* is obtained with the following equation: 
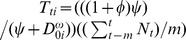
. The first part accounts for the proportion of the total traffic over the bridge that passes over the body of the ant *i* and depends on 3 control parameters *φ*, *ψ* and *ω* (see below for the estimation of their respective values), and on the packing density 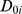 of ants in the bridge structure at the time where the ant *i* joined the bridge. Since they are more likely to occupy a central position in the bridge, ants that join when the packing density is low will tend to experience a larger proportion of the future traffic than the ants that join when the packing density is already high. The second part of the equation accounts for the ability of ants to integrate the traffic flow over their body for the last *m* seconds (*m* = 5, as suggested in [24]).

Only three parameters, *φ*, *ψ* and *ω*, could not be obtained directly from our experimental data. Their values were estimated by minimising the difference between the average dynamics of the number of ants forming the bridge in our experiments and in simulations of the model under similar conditions (bridge lengths and traffic intensity). The best fit was obtained for *φ*∼−0.52, *ψ*∼3 and *ω*∼2, with an average square difference between experimental and simulation data points of only 0.12 ants.

### Simulations

#### Effect of traffic intensity

For each value of λ, we ran 1000 simulations with duration of 600 seconds each. This duration corresponds to that between the destruction of a bridge in a given replicate of the bridge construction experiment and the start of the next replicate. For each value of λ, we computed the proportion of time during which at least one ant was stopped in the gap (non-empty bridge time ratio).

#### Effect of traffic oscillations

Traffic oscillations started from the beginning of a simulation (no ants filling the gap) and the simulation ended after 100 oscillation periods. 1000 replicate simulations were run for each tested combination of period and intensity. For each combination, we computed the non-empty bridge time ratio.

#### Response to sudden change in traffic intensity

Initial conditions (gap size and number of ants in the bridge) were set at the same values than those measured when trail sweeping started in the traffic perturbation experiment. The value of 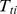 for each ant present in the bridge at the beginning of the simulation was computed with 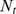 considered constant during the last m seconds and equal to the traffic intensity when trail sweeping started. To estimate the value of 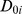 for each of these ants, we assumed that they joined the bridge sequentially, with the first one stopping as the gap was empty. At each time step of the simulation λ was set to match the traffic intensity measured at the corresponding time step in the traffic perturbation experiment.

## Supporting Information

Figure S1 Survival analysis of the time spent by ants as part of the bridge structure during the 2 minutes after the destruction of the bridge (main figure). A Cox Proportional Hazard Model reveals that this time increases with the overall packing fraction of the bridge (number of ants by unit length of the bridge, see inset). It also shows that ants joining the bridge earlier are less likely to leave it (not shown).
(TIF)

Figure S2 The traffic flow over an ant (a) and the number of surrounding ants in the structure (b) are both more important when the ant occupy a central position in the bridge rather than if it is stopped on the edge of it (Mann-Withney test, p = 0.0005 and p = 0.0164 respectively).
(TIF)

Figure S3 Comparison between measurements of total traffic flow by manual counting and optical flow analysis. Manual counting was performed on a total of 8 trails out of the 57 we recorded. We present here the results for 3 of these 8 trails (results are similar in the 8 trails). Each row represents: on the left, the normalized traffic flow (traffic flow divided by maximum traffic flow) measured by manual counting (black line) and by optical flow technique (red line); on the right, the cross-correlation between the manual and optical flow data with a maximum significant correlation for a time lag of zero second indicating a good match between manual and automated techniques.
(TIF)

Video S1 Video recording of a bridge removal experiment. The video shows the bridge structure and the traffic over the bridge before and after all ants in the bridge were removed with tweezers.
(MOV)

Video S2 Video recording of an army ant trail and demonstration of the optical flow analysis to estimate traffic variation on the trail.
(MOV)

## References

[1] Deisboeck TS, Couzin ID (2009) Collective behavior in cancer cell populations. Bioessays 31: 190–197 doi:10.1002/bies.200800084 19204991 10.1002/bies.200800084

[2] Szabó B, Szöllösi GJ, Gönci B, Jurányi Z, Selmeczi D, et al (2006) Phase transition in the collective migration of tissue cells: experiment and model. Phys Rev E Stat Nonlin Soft Matter Phys 74: 061908–061908 doi:10.1103/PhysRevE.74.061908 17280097 10.1103/PhysRevE.74.061908

[3] Friedl P, Gilmour D (2009) Collective cell migration in morphogenesis, regeneration and cancer. Nat Rev Mol Cell Biol 10: 445–457 doi:10.1038/nrm2720 19546857 10.1038/nrm2720

[4] Camazine S, Deneubourg J-L, Franks NR, Sneyd J, Theraulaz G, et al.. (2001) Self-organization in biological systems. Princeton, NJUSA: Princeton University Press.

[5] Couzin ID (2009) Collective cognition in animal groups. Trends in cognitive sciences 13: 36–43 doi:10.1016/j.tics.2008.10.002 19058992 10.1016/j.tics.2008.10.002

[6] Sumpter DJT (2010) Collective Animal Behavior. Princeton, NJUSA: Princeton University Press. 312 pp.

[7] Garnier S, Gautrais J, Theraulaz G (2007) The biological principles of swarm intelligence. Swarm Intelligence 1: 3–31 doi:10.1007/s11721-007-0004-y

[8] Couzin ID, Ioannou CC, Demirel G, Gross T, Torney CJ, et al (2011) Uninformed individuals promote democratic consensus in animal groups. Science 334: 1578–1580 doi:10.1126/science.1210280 22174256 10.1126/science.1210280

[9] Centola D (2010) The spread of behavior in an online social network experiment. Science 329: 1194–1197 doi:10.1126/science.1185231 20813952 10.1126/science.1185231

[10] Watts DJ, Dodds PS (2007) Influentials, Networks, and Public Opinion Formation. The Journal of Consumer Research 34: 441–458 doi:10.1086/518527

[11] Lewis K, Gonzalez M, Kaufman J (2012) Social selection and peer influence in an online social network. PNAS 109: 68–72 doi:10.1073/pnas.1109739109 22184242 10.1073/pnas.1109739109 PMC3252911

[12] Lind PG, da Silva LR, Andrade JSJ, Herrmann HJ (2007) Spreading gossip in social networks. Physical Review E 76: 36117 doi:10.1103/PhysRevE.76.036117 10.1103/PhysRevE.76.036117 17930316

[13] Helbing D, Molnár P (1998) Self-Organization Phenomena in Pedestrian Crowds. arXiv cond-mat.stat-mech 6152.

[14] Helbing D, Johansson A, Al-Abideen HZ (2007) Dynamics of crowd disasters: An empirical study. Physical Review E 75: 46109 doi:10.1103/PhysRevE.75.046109 10.1103/PhysRevE.75.046109 17500963

[15] Moussaïd M, Helbing D, Garnier S, Johansson A, Combe M, et al (2009) Experimental study of the behavioural mechanisms underlying self-organization in human crowds. Proc Biol Sci 276: 2755–2762 doi:10.1098/rspb.2009.0405 19439442 10.1098/rspb.2009.0405 PMC2839952

[16] Moussaïd M, Helbing D, Theraulaz G (2011) How simple rules determine pedestrian behavior and crowd disasters. PNAS 108: 6884–6888 doi:10.1073/pnas.1016507108 21502518 10.1073/pnas.1016507108 PMC3084058

[17] Couzin ID, Franks NR (2003) Self-organized lane formation and optimized traffic flow in army ants. Proc Biol Sci 270: 139–146 doi:10.1098/rspb.2002.2210 12590751 10.1098/rspb.2002.2210 PMC1691225

[18] Dussutour A, Fourcassié V, Helbing D, Deneubourg J-L (2004) Optimal traffic organization in ants under crowded conditions. Nature 428: 70–73 doi:10.1038/nature02344.1 14999281 10.1038/nature02345

[19] Dussutour A, Deneubourg J-L, Fourcassié V (2005) Temporal organization of bi-directional traffic in the ant Lasius niger (L.). J Exp Biol 208: 2903–2912 doi:10.1242/jeb.01711 16043595 10.1242/jeb.01711

[20] Burd (2006) Ecological consequences of traffic organisation in ant societies. Physica A 372: 8–8 doi:10.1016/j.physa.2006.05.004

[21] Rettenmeyer CW (1963) Behavioral studies of army ants. University of Kansas Publications 185.

[22] Schneirla TC (1972) Army Ants: A Study in Social Organization. Topoff HR, editor. W.H.Freeman & Co Ltd 369.

[23] Gotwald WH (1995) Army Ants: The Biology of Social Predation (Cornell Series in Arthropod Biology). Cornell University Press. 302 pp.

[24] Powell S, Franks NR (2007) How a few help all : living pothole plugs speed prey delivery in the army ant Eciton burchellii. Animal Behaviour 73: 1067–1076 doi:10.1016/j.anbehav.2006.11.005

[25] Franks NR (1985) Reproduction, foraging efficiency and worker polymorphism in army ants. In: Hölldobler B, Lindauer M, editors. Experimental behavioral ecology and sociobiology: in memoriam Karl von Frisch, 1886–1982. Sunderland, MAUSA: Sinauer Associates, Vol. 31: . pp. 91–107.

[26] Anderson C, Theraulaz G, Deneubourg J (2002) Self-assemblages in insect societies. Insectes Sociaux 49: 99–110 doi:10.1007/s00040-002-8286-y

[27] Mlot NJ, Tovey CA, Hu DL (2011) Fire ants self-assemble into waterproof rafts to survive floods. PNAS 108: 7669–7673 doi:10.1073/pnas.1016658108 21518911 10.1073/pnas.1016658108 PMC3093451

[28] Paragios N, Chen Y, Faugeras OD (2010) editors (2010) Handbook of Mathematical Models in Computer Vision. 1st ed. Springer 639.

[29] Wright C, Roberg P (1998) The conceptual structure of traffic jams. Transport Policy 5: 23–35 doi:10.1016/S0967-070X(98)00006-7

[30] Helbing D (2001) Traffic and related self-driven many-particles systems. Reviews of Modern Physics 73: 1067–1141 Available: http://link.aps.org/doi/10.1103/RevModPhys.73.1067

[31] Wrege PH, Wikelski M, Mandel JT, Rassweiler T, Couzin ID (2005) Antbirds Parasitize Foraging Army Ants. Ecology 86: 555–559 doi:10.1890/04-1133

[32] Hölldobler B, Wilson EO (1990) The ants. Cambridge, Mass.: Belknap Press of Harvard University Press.

[33] Kushner DJ (1969) Self-assembly of biological structures. Bacteriol Rev 33: 302–345. 4896352 10.1128/br.33.2.302-345.1969 PMC378323

[34] Whitesides GM, Grzybowski B (2002) Self-assembly at all scales. Science 295: 2418–2421 doi:10.1126/science.1070821 11923529 10.1126/science.1070821

[35] Halley JD, Winkler DA (2008) Consistent concepts of self-organization and self-assembly. Complexity 14: 10–17 doi:10.1002/cplx.20235

[36] Franks NR, Sendova-Franks AB, Simmons J, Mogie M (1999) Convergent evolution, superefficient teams and tempo in Old and New World army ants. Proc Biol Sci 266: 1697–1701 doi:10.1098/rspb.1999.0834

[37] Franks NR, Sendova-Franks AB, Anderson C (2001) Division of labour within teams of New World and Old World army ants. Animal Behaviour 62: 635–642 doi:10.1006/anbe.2001.1794

[38] Bonabeau E, Theraulaz G, Dorigo M (1999) Swarm Intelligence: From Natural to Artificial Systems. 1st ed. Oxford University Press, USA. 320 pp.

[39] Eberhart RC, Shi Y, Kennedy J (2001) Swarm Intelligence (The Morgan Kaufmann Series in Evolutionary Computation). 1st ed. Morgan Kaufmann 512.

[40] Sahin E (2005) Swarm robotics: From sources of inspiration to domains of application. In: Sahin E, editor. Swarm robotics. Vol. 3342. Springer. pp. 10–20. doi:10.1007/978-3-540-30552-1_2.

[41] Kennedy J (2006) Handbook of Nature-Inspired and Innovative Computing. Zomaya AY, editor Boston: Kluwer Academic Publishers. 33 pp. doi:10.1007/0-387-27705-6_6.

[42] Team RDC (2011) R: A Language and Environment for Statistical Computing. Vienna, Austria: R Foundation for Statistical Computing. Available: http://www.R-project.org/.

[43] Grambsch PM, Therneau TM (1994) Proportional Hazards Tests and Diagnostics Based on Weighted Residuals. Biometrika 81: 515–526.

[44] Glynn EF, Chen J, Mushegian AR (2006) Detecting periodic patterns in unevenly spaced gene expression time series using Lomb-Scargle periodograms. Bioinformatics 22: 310–316 doi:10.1093/bioinformatics/bti789 16303799 10.1093/bioinformatics/bti789

