# Supplementary figures and images for: Stability and Responsiveness in a Self-Organized Living Architecture
Source: PLoS Comput Biol. 2013 Mar 28;9(3):e1002984. doi: 10.1371/journal.pcbi.1002984 (PMC3610604; doi:10.1371/journal.pcbi.1002984)

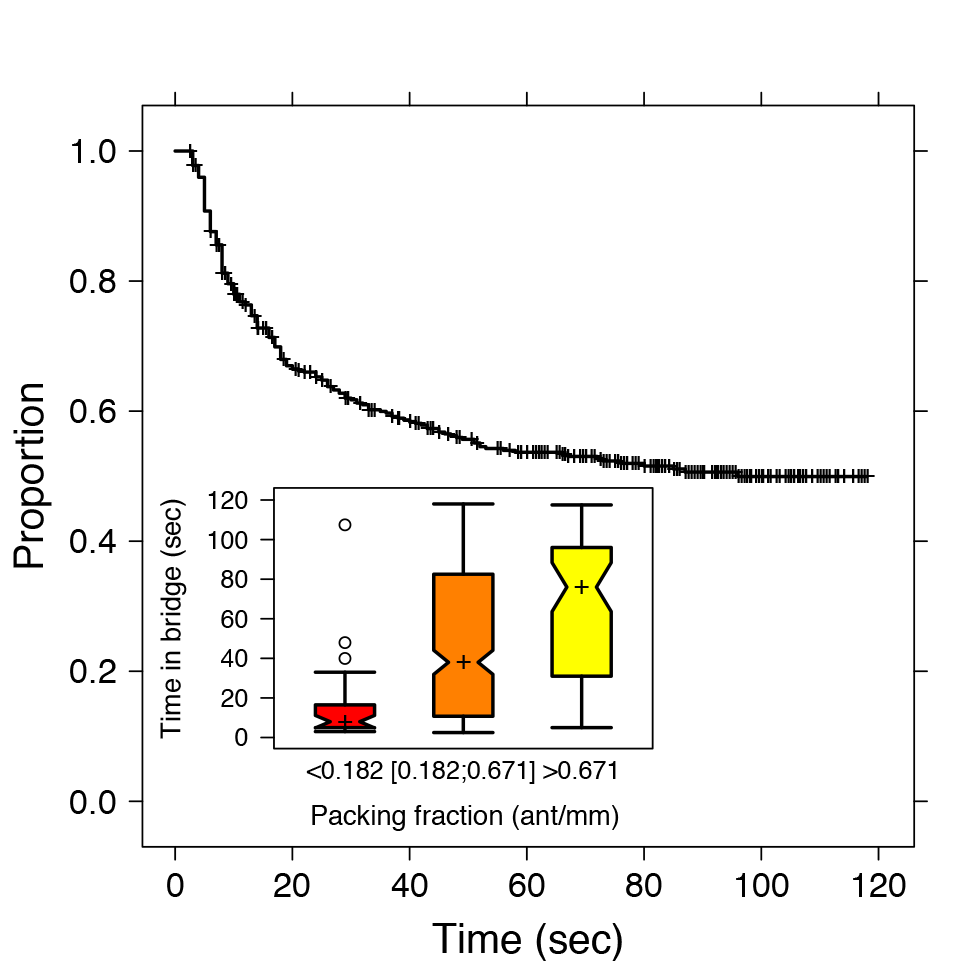

Supplement: Figure S1 — Survival analysis of the time spent by ants as part of the bridge structure during the 2 minutes after the destruction of the bridge (main figure). A Cox Proportional Hazard Model reveals that this time increases with the overall packing fraction of the bridge (number of ants by unit length of the bridge, see inset). It also shows that ants joining the bridge earlier are less likely to leave it (not shown). (TIF) [file pcbi.1002984.s001.tif]

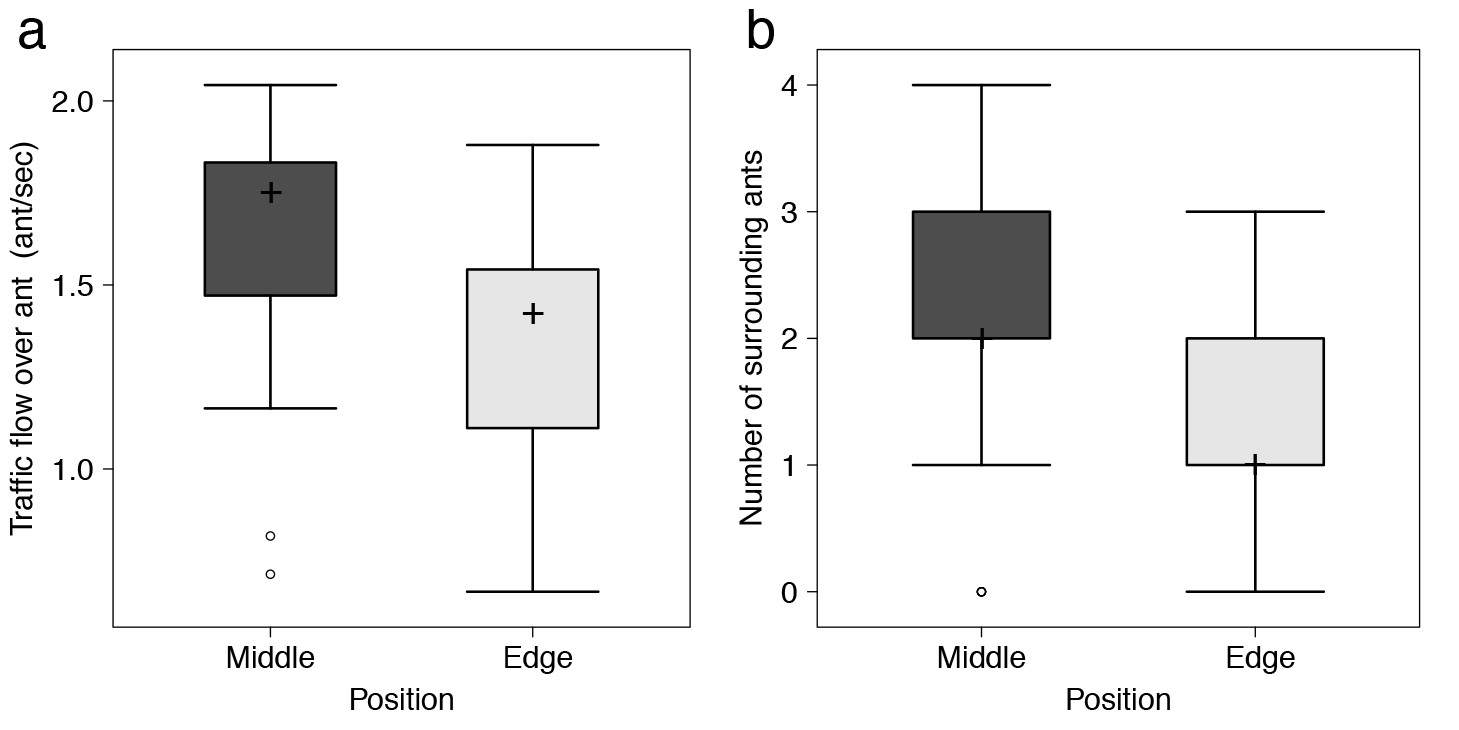

Supplement: Figure S2 — The traffic flow over an ant (a) and the number of surrounding ants in the structure (b) are both more important when the ant occupy a central position in the bridge rather than if it is stopped on the edge of it (Mann-Withney test, p = 0.0005 and p = 0.0164 respectively). (TIF) [file pcbi.1002984.s002.tif]

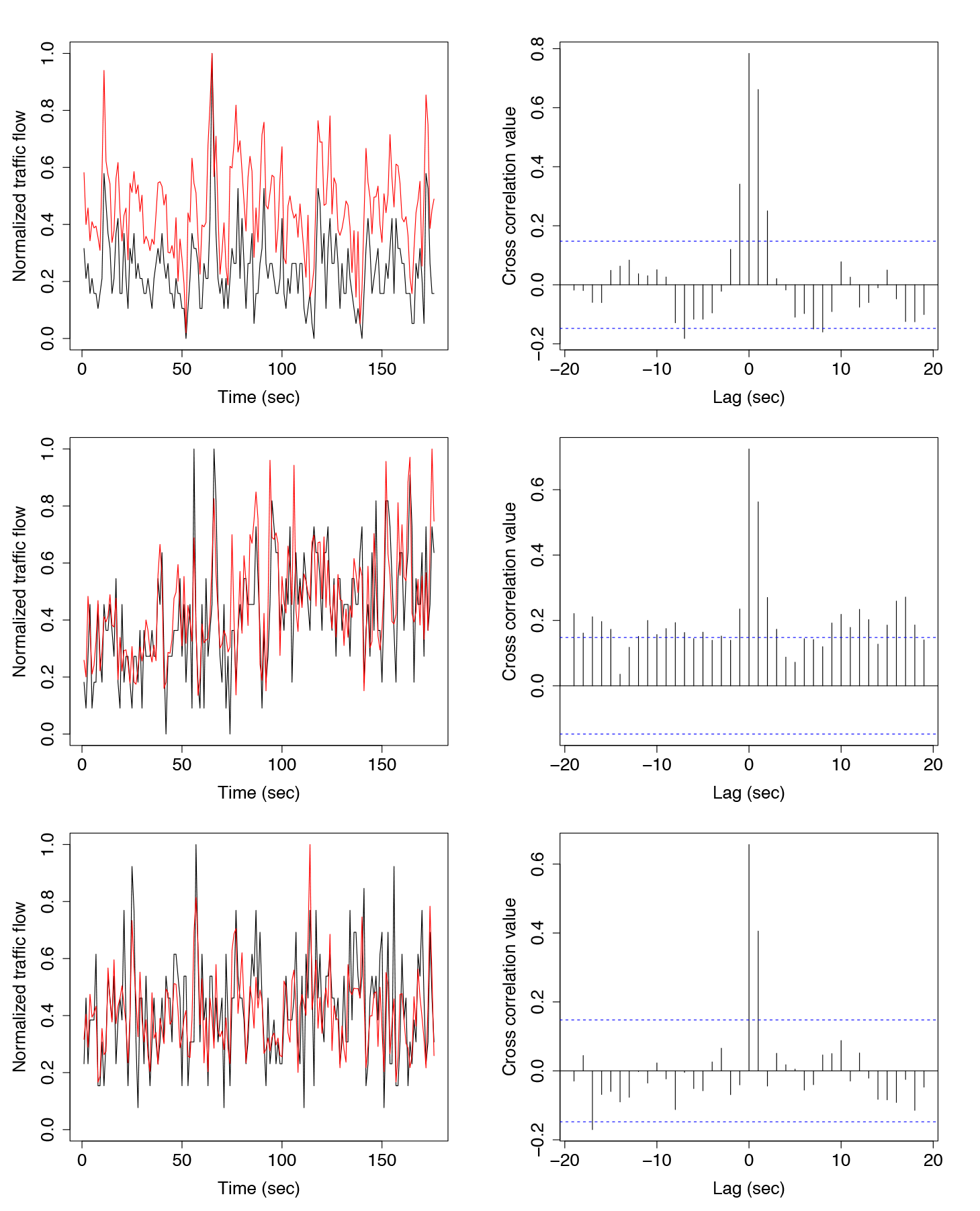

Supplement: Figure S3 — Comparison between measurements of total traffic flow by manual counting and optical flow analysis. Manual counting was performed on a total of 8 trails out of the 57 we recorded. We present here the results for 3 of these 8 trails (results are similar in the 8 trails). Each row represents: on the left, the normalized traffic flow (traffic flow divided by maximum traffic flow) measured by manual counting (black line) and by optical flow technique (red line); on the right, the cross-correlation between the manual and optical flow data with a maximum significant correlation for a time lag of zero second indicating a good match between manual and automated techniques. (TIF) [file pcbi.1002984.s003.tif]
